# Rugby Fans in Training New Zealand (RUFIT-NZ): protocol for a randomized controlled trial to assess the effectiveness and cost-effectiveness of a healthy lifestyle program for overweight men delivered through professional rugby clubs in New Zealand

**DOI:** 10.1186/s13063-019-4038-4

**Published:** 2020-02-04

**Authors:** Ralph Maddison, Elaine Anne Hargreaves, Yannan Jiang, Amanda Jane Calder, Sally Wyke, Cindy M. Gray, Kate Hunt, David Lubans, Helen Eyles, Nick Draper, Ihirangi Heke, Stephen Kara, Gerhard Sundborn, Claire Arandjus, Matthew Jenkins, Samantha Marsh

**Affiliations:** 1 National Institute for Health Innovation, University of Auckland, Auckland, New Zealand; 2 Institute for Physical Activity and Nutrition, Deakin University, Melbourne, VIC Australia; 3 School of Physical Education, Sport and Exercise Sciences, University of Otago, Dunedin, New Zealand; 4 Institute for Health and Wellbeing, College of Social Sciences, University of Glasgow, Glasgow, Scotland; 5 Institute for Social Marketing, Faculty of Health and Sports Sciences, University of Stirling, Stirling, UK; 6 School of Education, Priority Research Centre for Physical Activity and Nutrition University of Newcastle, Newcastle, NSW Australia; 7 National Institute for Health Innovation and Department of Epidemiology and Biostatistics, University of Auckland, Auckland, New Zealand; 8 School of Health Sciences, University of Canterbury, Christchurch, New Zealand; 9 Heke Consulting, Auckland, New Zealand; 10 Axis Sport Medicine Clinic, Auckland, New Zealand; 11 Department of Pacific Health, University of Auckland, Auckland, New Zealand

**Keywords:** Physical activity, Obesity, Weight loss, Men’s health, Lifestyle Intervention

## Abstract

**Background:**

A healthy lifestyle program that appeals to, and supports, obese New Zealand (NZ) European, Māori (indigenous) and Pasifika men to achieve weight loss is urgently needed. In Scotland, Football Fans in Training (FFIT), a weight management and healthy lifestyle program for overweight and obese men aged 35–65 years , delivered by community coaching staff at professional football clubs, has been shown to be beneficial and cost-effective. A pilot program inspired by FFIT but delivered by professional rugby clubs in NZ (*n* = 96) was shown to be effective in weight loss, improved physiological outcomes, and adherence to healthy lifestyle behaviors in overweight and obese men. The objective of this trial is to determine the effectiveness and cost-effectiveness of the Rugby Fans in Training New Zealand (RUFIT-NZ) program.

**Methods:**

A pragmatic, two-arm, multi-center, randomized controlled trial involving 308 overweight and obese men aged 30–65 years, randomized to either an intervention group (*n* = 154) or a wait-list control group (*n* = 154). The intervention-group participated in the 12-week RUFIT-NZ program, a gender-sensitized, healthy lifestyle intervention adapted to the environment and cultural diversity of NZ and delivered through professional rugby clubs. Participants in the intervention group undergo physical training sessions, in addition to workshop-based sessions to learn about nutrition, physical activity, sleep, sedentary behavior, and a range of behavior-change strategies for sustaining a healthier lifestyle. The control group receives the program after 52 weeks. The primary outcome is change in body weight from baseline to 52 weeks. Secondary outcomes include change in body weight at 12 weeks; waist circumference, blood pressure, fitness, and lifestyle behaviors at 12 and 52 weeks; and cost-effectiveness. A process evaluation informed by the RE-AIM framework will evaluate potential implementation of RUFIT-NZ as an ongoing program in NZ after the trial.

**Discussion:**

This trial will investigate the effectiveness and cost-effectiveness of the RUFIT-NZ program in overweight and obese NZ men.

**Trial registration:**

Australia New Zealand Clinical Trials Registry, ACTRN12619000069156. Registered on 18 January 2019, according to the World Health Organization Trial Registration Data Set. Universal Trial Number, U1111-1245-0645.

## Background

High Body Mass Index (BMI), poor diet, and physical inactivity were ranked among the top-10 risk factors attributable to the global burden of disease in 2010 [1]. In New Zealand (NZ), 32% of adults are obese (BMI > 30 kg/m^2^) and a further 35% are overweight, with notable sex and ethnic disparities. Compared with women, the prevalence of overweight is greater in NZ men classified as NZ European/Other (41% vs 32%), Māori (the Indigenous peoples of NZ; 33% vs 27%), and Pasifika (26% vs 16%, respectively) [2]. Further, Māori and Pasifika men are 1.7 and 2.2 times more likely to be obese when compared with non-Māori and non-Pasifika men, respectively. Clearly overweight and obese NZ men, and in particular Māori and Pasifika men, are underserved by existing public health strategies [3, 4]. Healthy lifestyle programs that are both appealing to, and support, these men in weight loss and long-term lifestyle changes are urgently needed.

In the United Kingdom, Football Fans in Training (FFIT), a weight management and healthy lifestyle program, was developed to specifically target overweight and obese (BMI 28 kg/m^2^ or above) middle-aged men (aged 35–65 years) [5, 6]. FFIT was delivered via professional soccer clubs in Scotland, in an attempt to appeal to men, and draw on their fandom or allegiance to the clubs [7–9]. FFIT is evidence-based, gender-sensitized in context, content and style of delivery, includes behavior-change techniques known to be effective in promoting weight loss and physical activity [10, 11] and includes components designed to improve healthy eating, physical activity, and alcohol consumption [12]. A randomized controlled trial (RCT) of FFIT (*n* = 748) showed a mean difference in weight loss of 4.94 kg (95% CI 3.95–5.94) at 12 months [5]. After 3.5 years, 65% of the men had maintained a mean weight loss of 2.9 kg [13]. Since the original FFIT trial, there has been considerable interest in using professional sports clubs to encourage men to participate in a range of health promotion initiatives [5, 14–20]. Qualitative research has found that the social support received through participating in these sport-based lifestyle programs is highly valued by participants [21–23]. However, the type of support provided, and the extent to which participants gain a sense of identity with the program, have not been investigated.

In NZ, where a dominant rugby culture exists with large fan bases among NZ European, Māori and Pasifika people, we developed and piloted Rugby Fans in Training New Zealand (RUFIT-NZ), a program inspired by FFIT, but where professional football clubs were replaced with professional rugby clubs to harness sports club affiliation [8]. Formative work conducted prior to the pilot study sought to determine the level of cultural adaptation required to engage Māori and Pasifika men in NZ and to meet the needs of NZ men more generally. This resulted in some adaptations to the UK FFIT program for NZ, including a more holistic perspective of health or “*haoura”* in Māori [24], and changes to workshop sessions to include mindful eating, improving sleep, and reducing screen use and sedentary behaviors. Finally, for RUFIT-NZ, nutrition information was delivered by a nutritionist, reflecting men’s desires to receive dietary advice from someone with specialist knowledge [25].

A pilot RCT (*n* = 96) was conducted to evaluate the preliminary efficacy of RUFIT-NZ and to address feasibility issues including recruitment, retention, and acceptability of the program. After the 12-week intervention, a − 2.5 kg (95% CI − 0.4 to 5.4) difference in body weight was observed in favor of the intervention group. In addition, participants who received the program had significant reductions in waist circumference, resting heart rate, diastolic blood pressure, as well as improved fitness and improved adherence to lifestyle behaviors, including not smoking, and being physically active [25]. Furthermore, 100% of those who completed the program said that they would recommend it to their friends, therefore supporting the feasibility and acceptability of RUFIT-NZ, and supporting a larger-scale RCT of the program [25].

This paper describes the study protocol for the RUFIT-NZ main trial, which aims to determine the effectiveness and cost-effectiveness of RUFIT-NZ on weight loss and improvements in diet, physical activity, alcohol use, and in perceived social support at 52 weeks in overweight men aged 30–65 years. A secondary aim is to undertake an embedded process evaluation of RUFIT-NZ to assess reach, effectiveness, adoption, implementation, and maintenance outcomes [26, 27]. Two dimensions operate at the individual level (reach and effectiveness) Additional file 1.

## Methods

### Study design

RUFIT-NZ is a pragmatic, multi-center, two-arm, parallel RCT, which includes an embedded process evaluation framework (RE-AIM) to assess reach, effectiveness, adoption, implementation, and maintenance outcomes. *R*each refers to the proportion and characteristics of participants who receive the intervention. *E*ffectiveness concerns the impact of the intervention on the intended outcome. Adoption and implementation operate at the staff and organizational level. *A*doption refers to the percentage and characteristics of staff and settings that are willing to adopt or implement the intervention. *I*mplementation refers to the fidelity of implementing the intervention across settings and staff. *M*aintenance refers to the sustainability of the intervention once research support has ceased. This protocol has been prepared in accordance with the Standard Protocol Items: Recommendations for Interventional trials (SPIRIT) 2013 Statement [28] and is presented in Table 1. The SPIRIT Checklist is shown as Additional file 2. Further, the intervention is described according to the Consolidated Standards of Reporting Trials (CONSORT) Checklist [29, 30].

**Table 1:**
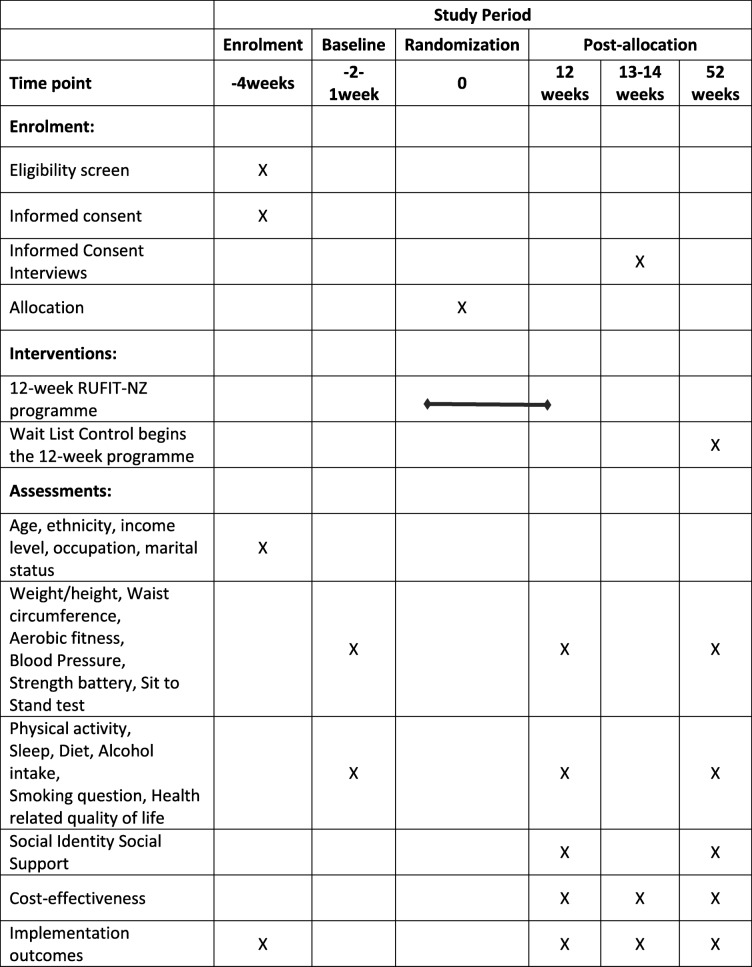
The schedule of the Rugby Fans in Training New Zealand (RUFIT-NZ) study period including; enrollment, interventions, and assessments

### Study setting

RUFIT-NZ is based within professional rugby clubs who participate in the Super Rugby competition across NZ (currently The Blues rugby club based in Auckland (North Island), The BNZ Crusaders based in Christchurch, and the Pulse Energy Highlanders based in Dunedin (both in the South Island)).

### Study population and recruitment

Eligible participants are men aged 30–65 years who are overweight (defined as a BMI of ≥ 28 kg/m^2^), able to safely undertake physical activity, can understand and read English, and are able to provide written informed consent to participate in the study. All participants are pre-screened using the Physical Activity Readiness Questionnaire (PAR-Q) [31, 32] with physician consent to participate required for all participants endorsing any PAR-Q items. Participants are excluded if they are participating in another healthy lifestyle program or know at baseline that they will be unable to complete the 1-year follow-up. Participants are recruited via the respective club’s fan base registries, including their Facebook pages, supporter mailing lists, and newspaper advertisements/articles; they are also recruited through Māori-specific networks (via *Marae* and media (e.g., Māori television and radio)) and Facebook advertisements.

Each advertisement links to the University of Auckland’s Faculty of Medical and Health Science’s research study recruitment page. Using this page, participants access additional information about the study, the Participant Information Sheet/Consent Form, and have the option of contacting the research team for further information or linking directly to the online plan and registration form. As part of the screening questionnaire, participants confirm that they can attend one of the pre-specified baseline assessments located at their preferred rugby club, and complete the PAR-Q [31]. Participants are reminded to bring a letter to the baseline assessment from their general practitioner (GP) confirming that it is safe for them to exercise and participate in the study (if required). Once the online self-report screening questionnaire is complete, eligible participants are asked to select the pre-determined day and time that they would prefer to come to the club to complete their baseline measurements. During the baseline assessment and following collection of their weight and height, and calculation of BMI, participant eligibility is confirmed (see Additional file 3).

### Sample size

A total of 308 participants (154 per arm) will provide 90% power at 5% significance level (two-sided) to detect a clinically significant 5-kg difference [7] on the primary outcome between the two groups at 52 weeks, assuming a standard deviation (SD) of 12 kg allowing for 20% loss to follow-up. Our SD is conservative and was derived from similar weight management trials for men [25, 33]. Based on the pilot study, the proposed 5-kg difference in body weight would be similar to a reduction in body mass of between 4 and 5% [25]. As the indigenous population in NZ, we aim to recruit a total of 150 Māori participants (~ 50% of the total sample size), which would provide 80% power to detect a 6-kg difference between groups under the same assumptions. Each club will implement strategies to minimize loss to follow-up and improve retention throughout the intervention (i.e., encouraging attendance, trainers notifying the coordinating center if men are absent for more than 2 weeks in a row with no explanation, and social media groups that all the men join) and for the follow-up testing sessions (i.e., provide the men with club merchandise where available, have club players present at the session, and offer alternate session times if required). As part of the consent process, participants are informed that they may withdraw from the program at any time during the study, without providing a reason to the research team or club.

### Randomization

Following baseline data collection, eligible and consented participants are randomized to either the RUFIT-NZ intervention or the control group in a 1:1 ratio using a computerized randomization process, ensuring allocation concealment. Randomization is stratified by baseline BMI category (< 35 kg/m^2^ vs ≥ 35 kg/m^2^), self-reported ethnicity (Māori, Pasifika, non-Māori/non-Pasifika), and study center, using stratified block randomization with variable block sizes of 2 or 4. Participants are informed by email of their eligibility and allocation group within 2–3 days of their baseline assessment at the club. Due to the nature of the study, participants and research assistants will be aware of the treatment allocation post randomization. Study investigators and trial statistician will remain blinded until the end of the trial. To reduce assessment bias, the primary and key secondary outcomes will be measured objectively at 12 and 52 weeks.

### Intervention

Specific details of the RUFIT-NZ program and how it was developed have been reported elsewhere [25]. In short, RUFIT-NZ is a 12-week healthy lifestyle program, consisting of 12 × 2-h sessions. Each intervention session includes a 1-h workshop-based education component (see Table 2) and 1-h group-based but individually tailored exercise training session. The education component aims to introduce participants to theory-based behavior-change techniques and to a range of topics relating to nutrition, sleep, alcohol consumption, and physical activity. The content of the classroom sessions has been standardized, so that the men participating at all clubs receive the same topic and key delivery points, but each club is able to tailor the sessions for delivery.

**Table 2:** The content of each workshop in the Rugby Fans in Training New Zealand (RUFIT-NZ) program

Week/session	Topics/core messages to cover	Facilitator
Week 1: Lifestyle approach to health and goal-setting	• Welcome and getting to know each other • Focus on lifestyle behaviors vs weight • SMART goal-setting • Team photo	Trainer
Week 2:*“Big wins” – nutrition*	• Whole food philosophy including social aspects • Big wins for healthy diets • Incorporating fruit and vegetables • Healthy drink options • Healthy serving sizes • Reading food labels	Nutritionist
*Week 3: Planning and budgeting – nutrition*	• Menu-planning • Budgeting • Shopping • Being organized/importance of routine	Nutritionist
Week 4: Behavior change	• Discussion of important behavior-change techniques: autonomous motivation, building confidence, goal setting, action and coping planning, self-monitoring, social support	Trainer
Week 5: *Mindful eating – nutrition*	• Focusing on your “circle of influence” • Eating out – mindful eating	Nutritionist
Week 6: Injury prevention	• Informal session designed by trainer to meet individual needs of men in team	Trainer/physio
Week 7: *Q & A – nutrition*	• Q & A session	Nutritionist
Week 8: Alcohol and health	• Alcohol weight-related facts • Standard drink sizes • Planning your drinking	Trainer
Week 9: Q & A (physical activity)	• Q & A session	Trainer
Week 10: Sleep and sedentary behavior	• How sleep affects weight • How much sleep we need • Signs of sleep deprivation • Sleep hygiene tips • What is sedentary behavior • How SB affects weight • Tips for reducing SB	Trainer
Week 11: Long-term behavior change and overcoming obstacles	• Importance of enjoying PA for long-term maintenance • Overcoming obstacles • Planning for lifestyle change • Relapse prevention	Trainer
Week 12: Team photo and motivational talk	• Wrap-up • Motivational talk • Team photo and certificate	Trainer

*SB* sedentary behavior, *PA* physical activity

Education sessions are delivered predominantly by RUFIT-NZ-trained trainers, with the exception of the nutrition-based components, which are delivered by the club’s nutritionist. RUFIT-NZ trainers are qualified strength and conditioning trainers involved with the respective rugby clubs.

With respect to the group-based in-stadia physical activity sessions, trainers are given the freedom to decide how to structure each of the sessions. This allows the trainers to decide how best to meet the needs of the individual men attending their specific RUFIT-NZ session. Each session progressively increases in difficulty over the 12 weeks, whilst taking account of each individual man’s levels of fitness and is designed to be fun and varied, utilizing the supportive group involvement to foster the sense of being in a “team.” The proposed group size ranges from approximately 15–20 men per trainer. Roll calls are taken at the beginning of each session to assess program attendance.

Throughout the intervention, men are encouraged to follow a daily step-based walking program [34–36]. All participants are encouraged to use a step counter (pedometer or smartphone app) to track their progress and receive a weekly step goal program to follow outside of the structured program. Furthermore, they are encouraged to do other forms of physical activity of their own choosing with a focus on integrating incidental activity into daily life (e.g., parking further from work). The RUFIT-NZ trainers also suggest “homework” physical activity that participants can do outside of the sessions. Participants’ lifestyle behaviors in terms of alcohol, sleep, sedentary behavior, and nutrition will be guided by individual goals, which men set for themselves during the group education sessions and record in a workbook accompanying the program for their own reference.

The RUFIT-NZ nutrition-based content was developed by our investigator nutritionist (HE), and is delivered using a Family, Activity, Behavior (FAB) approach [37]. However, because the diets of participants are influenced by their partners, family members (*whanau*) and friends, and the environment in which they live, learn, work, and play, the focus is on dietary changes that each participant can make within his own unique ‘circle of influence’ [38]. The nutrition sessions are positively framed (e.g., “what are some good examples of healthy snacks?” and “where can I find quick easy recipes?”). Information is delivered via simple messages focusing on the practical elements of improving diet, and where possible is food – rather than nutrient-focused. RUFIT-NZ nutrition sessions target the biggest “wins” for a healthy diet, considered to be:

- Eating as many fruit and vegetables as possible
- Cooking and preparing food and snacks at home as much as possible
- Eating mostly whole foods (as opposed to packaged/processed foods and takeaways)
- Drinking sugar-free beverages
- Conscious eating (screen-free, mindful eating, ideally in the company of others)

By focusing on these positive behaviors participants will, by default, consume less fast food, sugar-sweetened beverages, alcohol, energy, salt, sugar, and smaller portion sizes, reduce their risk of common nutrition-related diseases, and hopefully reduce their body weight. The men are also provided with a food diary to record their diet should they wish during the intervention. These messages also align with the NZ Eating and Activity Guidelines for Adults [39].

### Behavior-change techniques

A wide range of evidence-based behavior-change techniques is used throughout the education and exercise sessions to equip men with the skills to initiate and maintain a healthy lifestyle [40]. These are: (1) goal setting for, and self-monitoring of, weight, physical activity and healthy diet; (2) intention formation with action plans; (3) experiencing exercise sessions with increased challenges as well as positive feedback on exercise achievements and change reinforcement from trainers to build self-efficacy; and (4) identification of barriers and coping planning to help avoid relapse on completion of the program.

### Wait-list controls

The control group will continue with their usual lifestyle for 52 weeks during the trial period but will be offered the RUFIT-NZ program at the end of the 12-month follow-up period.

### Training

All RUFIT-NZ trainers undergo a standardized training session prior to delivering the program. The training is delivered by a member of the RUFIT-NZ investigator team. Trainers receive a standardized booklet outlining the key principles of the program, content details for each of the education sessions, including PowerPoint presentations, participant worksheets and resources to help facilitate group discussions and interactions. Trainers are introduced to the Supportive, Active, Autonomous, Fair, and Enjoyable (SAAFE) delivery principles, which is an evidence-based framework designed to guide the planning, delivery, and evaluation of organized physical activity sessions [41]. Trainers are encouraged to: (1) create a *S*upportive social environment, enabling learning from each other; (2) maximize participants’ opportunities to be physically *A*ctive during the sessions; (3) satisfy participants’ need for *A*utonomy by including elements of choice and providing a rationale for activities; (4) design and deliver experiences that are *F*air by allowing all participants to experience success regardless of their physical abilities; and (5) promote an *E*njoyable experience by focusing on fun and variety and incorporating games where possible.

Nutritionists are provided with a standardized document outlining the key principles to be promoted via RUFIT-NZ and the nutrition topics to be covered in each 1-h session. PowerPoint presentation templates, participant worksheets and resources are also offered to nutritionists to provide the basis for group discussions.

### Baseline demographic information

A self-report web-based questionnaire will be administered at baseline to gather demographic information (age, date of birth, ethnicity, employment status, highest level of education, marital status, and income) from all participants.

### Primary and secondary outcomes

#### Anthropometrics (baseline, 12- and 52-week follow-ups

(1) body weight (measured using calibrated digital scales, (2) waist circumference measured using a tape measure, and (3) height measured using a stadiometer. The primary outcome is defined as the change in body weight from baseline to 52 weeks.

#### Seated systolic and diastolic blood pressure (baseline, 12- and 52-week follow-ups)

Measured using an automated sphygmomanometer (OMRON T9P Intellisense Blood Pressure Monitor) and/or a manual (Reister) Blood Pressure Monitor.

#### Fitness (baseline, 12- and 52-week follow-ups)

Assessed by a 6-km cycle test, a timed sit-to-stand test, and a timed push-up test.

#### Lifestyle behaviors (baseline, 12- and 52-week follow-ups)

Assessed using a self-report questionnaire: (1) leisure time activity (assessed by the Godin Leisure Time Physical Activity Questionnaire) [42]; (2) cigarette smoking (assessed by a smoking history questionnaire) [43]. (3) alcohol intake (assessed by the Alcohol Use Disorders Identification test consumption (AUDIT-C)) [44]; (4) sleep (assessed by the number of hours slept over a 24-h period); and (5) fruit and vegetable intake, discretionary foods, and sugar-sweetened beverage consumption (assessed by the NZ Health Survey questions) [45].

#### Social support and social identity (12- and 52-week follow-ups)

Assessed in a subgroup of participants using a modification of the Athlete Received Support Questionnaire (ARSQ) [46], and social identity measure, respectively [47].

#### RUFIT cost-effectiveness (baseline, 12- and 52-week follow-ups)

Assessed by the EQ-5D [48], a generic and validated measure of choice for which reliable NZ population preference values are available [48–50], to obtain a single preference index for calculation of Quality-Adjusted Life-Years (QALYs).

#### Implementation of RUFIT-NZ as an ongoing program (12- and 52-week follow-ups)

Assessed by an embedded process evaluation conducted using the RE-AIM framework [26, 27]. A mixed-methods (quantitative and qualitative) approach [51] will be used for the process evaluation (see Table 3 for specific details).

**Table 3:**
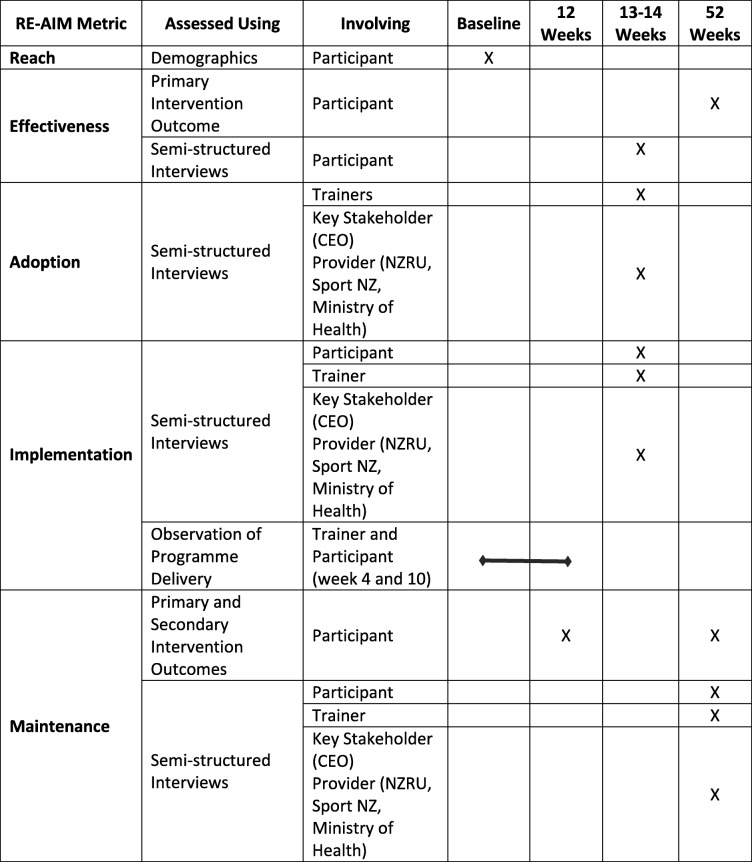
Process evaluation conducted using the Reach, Effectiveness, Adoption, Implementation, Maintenance (RE-AIM) metrics

### Data collection and management

All self-reported data are directly input via online surveys to the web-based data capture system Research Electronic Data Capture (REDCap) [52, 53] hosted at the National Institute for Health Innovation (NIHI). All objective data are collected on hardcopy at face-to-face sessions by trained researchers and then input to the same REDCap web-based data capture system hosted and managed at NIHI. At least two measurements are completed for each of the anthropometric and blood pressure measures. Anthropometrics, blood pressure and fitness testing data were collected by trained research assistants. All data are kept for 6 years and will only be used in future studies following a process of participant informed consent.

### Post-trial care

As this is a pragmatic trial, there are no post-trial plans for participants after the 52 weeks. Serious harm is not expected to arise as a result of the intervention. All trainers are required to have a current first-aid certificate and are instructed to report any serious adverse or adverse events related to exercise arising over the 12-week intervention to the central coordinating center. Participants are covered by the Accident Compensation Corporation (New Zealand’s no-fault accidental injury compensation scheme) in the unlikely event that they sustain a physical injury.

### Fidelity

Intervention fidelity is evaluated by means of direct observation by trained research assistants, using a standardized checklist at weeks 4 and 10 of the 12-week intervention. Feedback will be provided by the research team to the trainers to address any gaps in intervention delivery.

### Monitoring and auditing

A data monitoring committee was not deemed necessary due to the low-risk nature of the trial. Data are checked for any values that fall outside the range checks within the REDCap forms by the central coordinating center. An internal audit will be conducted on the trial by a senior project manager independent to the trial.

## Analysis

### Statistical effectiveness analysis

All participants’ data are collected via secure, web-based, case record forms developed by NIHI using REDCap. Statistical analysis will be performed using SAS version 9.4 (SAS Institute Inc., Cary, NC, USA). Baseline characteristics of all randomized participants (including age, ethnicity, employment status, highest level of education, marital status, and income) will be summarized descriptively for intervention and control groups separately. Continuous variables will be presented as means and standard deviations (SD). Categorical variables will be presented as frequencies and percentages. The results will be presented overall and by ethnic group.

Intervention evaluation will be performed on the principle of intention-to-treat (ITT). All statistical tests will be two-sided at a 5% significance level. The primary and secondary outcomes will be first summarized descriptively by randomized group at 12 and/or 52 weeks. The distribution of outcome measures will be checked. The primary outcome at 52 weeks will be evaluated using the analysis of covariance model, adjusting for baseline outcome value and stratification factors. Model-adjusted means and the difference between two groups will be estimated with 95% confidence interval and *p* value. Multiple imputations will be used on missing primary outcome data in the main ITT analysis. Sensitivity analyses using different assumptions on the missing data, including complete case analysis, will also be conducted to test the robustness of the main trial findings. Per-protocol analysis will be performed on randomized participants with no major protocol violations.

Generalized linear regression models will be used to analyze secondary outcomes measured at 12 and/or 52 weeks, using a link function appropriate to the distribution of outcome variable. The regression model will adjust for baseline outcome value (if measured) and stratification factors, similar to the primary analysis. Model-adjusted estimates will be presented with 95% confidence intervals and *p* values. For repeated outcome measures, random-effect mixed models will be used to take into account the correlated data collected from the same participant with a random-subject effect. The consistency of intervention effects between different ethnic groups will be tested using an interaction term between treatment group and ethnicity. As a pre-planned subgroup analysis, separate analyses will also be conducted and presented for the ethnic groups outlined above. Between-subjects’ analysis will compare differences in the mean social identity and social support scores between control- and intervention-group participants at 12 and 52 weeks. Within-subjects’ analysis will also be conducted to compare change in mean scores within groups at 12 and 52 weeks. No interim analysis is planned for the trial. The final trial results will be reported according to the Consolidated Standards of Reporting Trials (CONSORT) 2010 guidelines.

### Cost-effectiveness analysis

This analysis will adopt a healthcare perspective. The within-trial analysis will determine the cost-effectiveness of RUFIT-NZ compared with a non-active intervention. No intervention will be used as the comparator as, in the absence of the RUFIT-NZ, this is the most likely alternative for this population. Throughout the analyses, the wait-list comparison group will be used as the source of data for the “no active intervention” group. As an ongoing program, Markov modeling will combine data from RUFIT-NZ with other information from a systematic evaluation of cost-effectiveness studies of weight management interventions [54] to identify the long-term cost-effectiveness of the intervention.

### RE-AIM analysis

Qualitative data collection and analysis will be conducted by trained and experienced qualitative researchers. Interviews will be digitally recorded and transcribed. Data will be entered using NVivo11 software to facilitate qualitative analysis. An inductive thematic analysis approach will be used to identify the key themes emerging from the data [55, 56], which will then be collated according to the various RE-AIM principles [26, 27]. All RE-AIM data sources (see Table 3.) will be combined so that the Study Advisory Group and investigators will make informed recommendations to determine the extent to which RUFIT-NZ achieved the desired RE-AIM outcomes.

## Discussion

NZ men, and in particular Māori and Pasifika men, are at increased risk for overweight and obesity, but are a difficult group to engage with in healthy lifestyle programs [3, 4]. Findings from our previous feasibility work demonstrated that delivery of a healthy lifestyle program through professional rugby clubs is both feasible and acceptable to overweight NZ men [25]. If effective, the RUFIT-NZ program, therefore, offers an opportunity to reach NZ men at increased risk of ill-health. This paper reports the design of a RCT designed to assess the long-term effectiveness and cost-effectiveness of the RUFIT-NZ program, and to evaluate the potential for implementation of RUFIT-NZ as an ongoing program. If effectiveness and cost-effectiveness is demonstrated, RUFIT-NZ may prove to be a unique way to target overweight NZ men.

## Trial status

Trial recruitment started in January 2019 and 200 participants (65%) were randomized as of 21 June 2019, protocol version 2.0 dated: 11 April 2019.

## Supplementary information

**Additional file 1.** RUFIT-NZ Trial Registration Data.

**Additional file 2.** SPIRIT 2013 Checklist: Recommended items to address in a clinical trial protocol and related documents.

**Additional file 3.** Flow Chart Illustrating Enrolment and Randomisation Process.

**Additional file 4.** Participant Information Sheet and Consent Form.

**Additional file 5.** Award letter for study grant from the Health Research Council.

## Data Availability

No individual participant data will be shared from this trial.

## Abbreviations

ARSQ: Athlete Received Support Questionnaire
AUDIT C: Alcohol Use Disorders Identification test Consumption
BMI: Body Mass Index
CONSORT: Consolidated Standards of Reporting Trials
FAB: Family, Activity, Behavior
FFIT: Football Fans in Training
GP: General practitioner
ITT: Intention to treat
NIHI: National Institute for Health Innovation
NZ: New Zealand
PAR-Q: Physical Activity Readiness Questionnaire
QALYS: Quality-Adjusted Life-Years
RCT: Randomized controlled trial
RE-AIM: Reach, Effectiveness, Adoption, Implementation, Maintenance
REDCap: Research Electronic Data Capture
RUFIT-NZ: Rugby Fans in Training New Zealand
SAAFE: Supportive, Active, Autonomous, Fair, and Enjoyable
SD: Standard deviation
