# Supplementary material for: Rugby Fans in Training New Zealand (RUFIT-NZ): protocol for a randomized controlled trial to assess the effectiveness and cost-effectiveness of a healthy lifestyle program for overweight men delivered through professional rugby clubs in New Zealand
Source: Trials. 2020 Feb 4;21:139. doi: 10.1186/s13063-019-4038-4 (PMC7001306; doi:10.1186/s13063-019-4038-4)
Supplement: Supplementary file 1 — Additional file 1. RUFIT-NZ Trial Registration Data. [file 13063_2019_4038_MOESM1_ESM.docx]

| **RUFIT-NZ Trial Registration Data** | |
| --- | --- |
| **Data Category** |  |
| **Primary Registry and Trial Identifying Number** | Australia New Zealand Clinical Trials Registry  ACTRN12619000069156 |
| **Date of Registration in the Primary Registry** | 18 January, 2019 |
| **Secondary Identifying Numbers** | UTN: U1111-1245-0645 |
| **Source of Monetary or Material Support** | Health Research Council |
| **Primary Sponsor** | University of Auckland |
| **Secondary Sponsor** | University of Otago |
| **Contact for Public Queries** | Amanda Calder  0272679071  [rufit@auckland.ac.nz](mailto:rufit@auckland.ac.nz) |
| **Contact for Scientific Queries** | Principle Investigator: Ralph Maddison  (09) 373 7599  [r.maddison@auckland.ac.nz](mailto:r.maddison@auckland.ac.nz)  Co-Investigator: Elaine Hargreaves  (03) 479 8941  [Elaine.hargreaves@otago.ac.nz](mailto:Elaine.hargreaves@otago.ac.nz)  Research Fellow: Samantha Marsh  [sam.marsh@auckland.ac.nz](mailto:sam.marsh@auckland.ac.nz) |
| **Public Title** | RUgby Fans In Training (RUFIT-NZ): a healthy lifestyle programme for overweight men aged 30-65 years. |
| **Scientific Title** | RUgby Fans In Training (RUFIT-NZ): A randomized control trial to determine the effectiveness and cost-effectiveness of a healthy lifestyle programme on bodyweight in overweight men aged 30-65 years. |
| **Countries of Recruitment** | New Zealand |
| **Health Condition(s) or Problem(s) Studied** | Overweight and/or obesity  Sedentary Behaviour  Public Health  Health promotion/education  Diet and Nutrition  Obesity |
| **Intervention(s)** | 12-week RUFIT-NZ intervention programme: A multi-center, two-arm, parallel randomized controlled trial designed to assess effectiveness of the RUFIT-NZ programme, with respect to bodyweight, diet, physical activity, and alcohol use. The programme will involve a group-based healthy living and weight loss programme, delivered in 12 x weekly two-hour sessions at the Rugby club’s training facility, by a trained RUFIT coach. Each two-hour session will cater for approximately 20 people and include a workshop-based education component, the content of which will include behavior change (goal setting, problem solving, planning and budgeting, self-monitoring) for nutrition diet and exercise delivered by rugby club coaches The rugby coaches and nutritionists will be trained in the intervention and given slides, manuals, and seminar on the content for the education component. Each participant will receive a booklet containing the slides presented and forms to monitor their step-counts, weekly food consumption, weight, and their weekly goals. The individually-tailored exercise training programmes are strength and conditioning based delivered and prepared by the rugby club coaches. Coaches will create programmes for all participants to complete but that cater to individual fitness abilities. For example, if the group is required to run the coaches will encourage those whose abilities do not meet those requirements to walk. Adherence to the intervention will be monitored through measuring bodyweight, diet, physical activity, and alcohol use at baseline, 12 weeks, and 52 weeks. Participants will be randomized to either the, 12-week RUFIT-NZ intervention programme or the wait-list control group who get the intervention after the 52 week measurements. |
| **Key Inclusion and Exclusion Criteria** | Inclusion Criteria: Men aged between 30-65 years who are overweight (defined as having a BMI 28kg/m^2^ or higher), can speak and write in English, give informed electronic consent, and who meet the Physical Activity Readiness Questionnaire criteria or (if not) provide consent from their physician to participate in the study.  Exclusion Criteria: Participants who are currently participating in another healthy lifestyle programme or are terminally unable to complete the 1-year duration of the intervention. |
| **Study Type** | Interventional  Allocation: Randomized controlled trial using a 1:1 ratio performed by computer with stratified block randomization based on baseline BMI category, self-reported ethnicity (Māori vs. non-Māori), and study center with variable block sizes of 2 or 4  Assignment: Multi-center, two-arm, parallel assignment  Purpose: Treatment |
| **Date of First Enrollment** | 21/01/2019 |
| **Sample Size** | Target Sample Size: 308  Current Sample Size: 200 |
| **Recruitment Status** | Recruiting |
| **Primary Outcome** | Body Weight: Assessed by comparing the weight of the intervention group to the control group measured by a digital weighing scale at baseline and 52 weeks post intervention start date. |
| **Key Secondary Outcomes** | All the below secondary outcomes are measured at Baseline, 12- and 52- weeks post-intervention start date unless otherwise specified.  Body Weight: Assessed by comparing the weight of the intervention group to the control group measured by a digital weighing scale at baseline and the 12-week follow-up.  Waist Circumference: Assessed objectively using standard procedures with a tape measure. Blood Pressure: Measured using an electronic blood pressure cuff.  Aerobic Fitness: Assessed by timing the duration of a 6km cycle ergometer test.  Strength Battery: Assessed by counting the number of press-ups completed in 60 seconds.  Sit to stand test: Assessed by counting the number of times the participants move from sitting to standing in 30 seconds.  Physical Activity: Measured objectively using Acti-graph accelerometers worn for seven days (in a sub-group only), and subjectively through the Godin Leisure Time Questionnaire which assesses the duration and frequency of strenuous, moderate, and light activity over the previous seven days.  Sleep: Measured by questions from the NZ Health Survey.  Diet: Assessed by the NZ Health Survey which measures consumption of fruit, vegetable, discretionary foods, and sugar sweetened beverages over the last seven days.  Alcohol Intake: Assessed using the Alcohol Use Disorders Identification Test to calculate the number of alcohol units consumed per week. Smoking: Assessed by questioning the participants smoking habits over the previous year.  Social Support and Social Identity: Assessed using a modification of the Athlete Received Support Questionnaire (ARSQ), and social identity measure respectively at 12- and 52-weeks post intervention start date. Implementation Potential: Assessed by using the RE-AIM framework which collects information on the Reach, Effectiveness, Adoption, Implementation, and Maintenance of an intervention through a combination of the measures described above and through semi-structured interviews with a sub-scale of participants and coaches from RUFIT-NZ after completion of the intervention.  Health-related quality of life: Measured using the New Zealand EQ-5D Tariff 2.  Serious Adverse Events (SAE): Recorded throughout the intervention if, and when they occur.  Cost-effectiveness: Assessed by calculating the direct cost of the programme through health care utilization, Ministry of Health datasets, and personal costs at 52 weeks post intervention start date. |
| **Ethics Review** | Status: Approved  Date: 20/09/2018 |
| **Completion Date** | Anticipated date of last data collection: 28 February 2021 |
| **Summary Results** | Analyses have not been conducted yet. |
| **IPD sharing statement** | No individual participant data will be shared from this trial |
