# Supplementary figures and images for: Rugby Fans in Training New Zealand (RUFIT-NZ): protocol for a randomized controlled trial to assess the effectiveness and cost-effectiveness of a healthy lifestyle program for overweight men delivered through professional rugby clubs in New Zealand
Source: Trials. 2020 Feb 4;21:139. doi: 10.1186/s13063-019-4038-4 (PMC7001306; doi:10.1186/s13063-019-4038-4)

## Slide 1
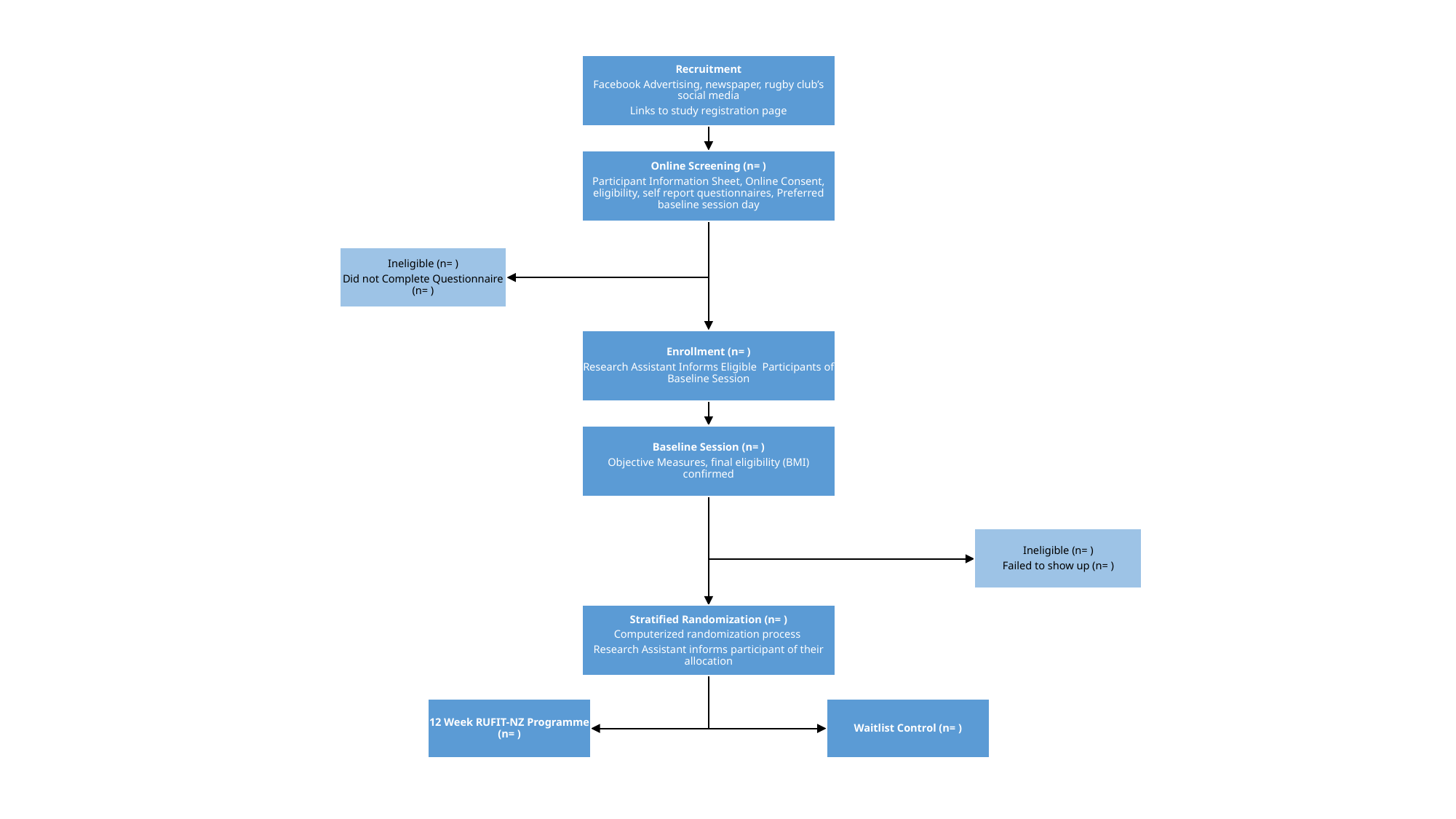

Supplement: Supplementary file 3 — Additional file 3. Flow Chart Illustrating Enrolment and Randomisation Process. [file 13063_2019_4038_MOESM3_ESM.pptx]
