# Supplementary material for: Rugby Fans in Training New Zealand (RUFIT-NZ): protocol for a randomized controlled trial to assess the effectiveness and cost-effectiveness of a healthy lifestyle program for overweight men delivered through professional rugby clubs in New Zealand
Source: Trials. 2020 Feb 4;21:139. doi: 10.1186/s13063-019-4038-4 (PMC7001306; doi:10.1186/s13063-019-4038-4)
Supplement: Supplementary file 4 — Additional file 4. Participant Information Sheet and Consent Form. [file 13063_2019_4038_MOESM4_ESM.pdf]

The National Institute for Health Innovation
School of Population Health
Tamaki Campus
The University of Auckland
Private Bag 92019, Auckland, New Zealand
Telephone: 09 923 4730

Participant Information Sheet

Rugby Fans In Training New Zealand (RuFIT-NZ)

Lead investigator: Professor Ralph Maddison
National Institute for Health Innovation, University of Auckland
08003676444
r.maddison@auckland.ac.nz

Researchers: Professor Ralph Maddison, Dr Elaine Hargreaves, Dr Samantha Marsh, Dr Ihirangi Heke, Dr Stephen Kara, Dr Gerhard Sundborn, Dr Helen Elyes, Professor Kate Hunt, Professor Sally Wyke, Dr Cindy Gray, Professor Dave Lubans, Dr Yannan Jiang, Professor Nick Draper

---

You are invited to take part in Rugby Fans In Training New Zealand (RUFIT-NZ), a healthy lifestyle programme for male rugby fans. The programme is being delivered through professional rugby clubs throughout New Zealand.

The study is randomised, which means you will either take part in the programme at the beginning of the study, or have the option of taking part in the programme at the end of the study (after 1 year).

To help you make a decision about participating in the study, we ask that you read this information sheet. Before you decide, you may want to talk about the study with other people, such as family, whanau, friends, or healthcare providers. Feel free to do this.

Who is undertaking the research?

This study is being undertaken by researchers at the National Institute for Health Innovation (University of Auckland), and the University of Otago. The research is funded by a Health Research Council Project grant.

What is the purpose of the study?

We have developed a programme that promotes healthy lifestyle behaviours in overweight males who are fans of rugby. The programme is being delivered through professional rugby clubs, including the Auckland Blues and the Highlanders.

The National Institute for Health Innovation
School of Population Health
Tamaki Campus
The University of Auckland
Private Bag 92019, Auckland, New Zealand
Telephone: 09 923 4730

Who can take part in the study?

You can take part in this study if you:

- Are male
- Are overweight (a body mass index of ≥28 kg/m2)
- Are able to safely undertake physical activity
- Can understand and read English
- Are aged 30-65 years
- Are able to provide consent to take part

In addition, **if you answer 'Yes' to any of the following questions,** we will also need a letter from your doctor advising us that it is safe for you to take part in the RuFIT-NZ programme:

1. Has your doctor ever said that you have a heart condition and that you should only do physical activity recommended by a doctor?
2. Do you feel pain in your chest when you do physical activity?
3. In the past month, have you had chest pain when you were not doing physical activity?
4. Do you lose your balance because of dizziness or do you ever lose consciousness?
5. Do you have a bone or joint problem (for example, back, knee, or hip) that could be made worse by a change in your physical activity?
6. Is your doctor currently prescribing drugs (for example, water pills) for your blood pressure or heart condition? [If so, we will need a letter from your doctor saying it is ok for you to participate in the study before you can take part in the study]
7. Do you know of any other reason why you should not do physical activity?

Where will the study take place?

All visits will take place at the either the club's facilities or a nearby training facility.

What is involved if I take part?

We are inviting 308 men to take part in the study. Everyone will be in the study for 1 year. If after reading the study information you decide to take part, you will need to answer some online questions to determine your initial eligibility (final confirmation of eligibility will be made after we have measured your height and weight during a baseline session).

If you meet the initial eligibility criteria, then you will be asked to provide online consent to participate in the study, and complete an online questionnaire. The questionnaire includes demographic questions, and questions about your sleep, diet, leisure time activities, and alcohol intake. You will then be asked to select a time that suits you to come in for a baseline assessment to

The National Institute for Health Innovation
School of Population Health
Tamaki Campus
The University of Auckland
Private Bag 92019, Auckland, New Zealand
Telephone: 09 923 4730

have your height, weight, and blood pressure measured, and complete a fitness assessment. At this baseline session, your height and weight will be used to calculate your BMI and determine your final eligibility to participate in the study. If you are eligible, you will be randomised to one of two groups:

1. RUFIT-NZ: This group will participate in the RUFIT-NZ programme, which will commence immediately (i.e. during the following week).
2. Wait-list controls: This group will be given the opportunity to participate in the RUFIT-NZ programme in 1 years' time (i.e. after completion of the 1-year visit).

You will be informed via email of which group you have been randomised to within a few days of attending the baseline session. If randomised to the RUFIT-NZ group, you will return the following week to start the programme.

Regardless of which group you are randomised to, we will need you to provide data at three different time points:

1. Baseline (i.e. before the study starts)
2. 12 weeks (i.e. at the end of the RUFIT-NZ programme)
3. 1 year after baseline

At each time point, you will be asked to complete an online questionnaire (this can be done at home at a time that suits you), and you will need attend a session at the club/training facility to have your physical measures assessed. Each of the three visits will take approximately 1 hour.

RUFIT-NZ programme:
The RUFIT-NZ programme consists of a total of 12 sessions, delivered once-weekly over a period of 12 weeks. Each session will run for 2 hours.

Each 2-hour session will include an education component and an individually-tailored exercise training component. The education component will cover different topics relating to nutrition, alcohol consumption, and physical activity. The exercise component will be designed by the RUFIT-NZ coach and will progressively increase in difficulty over 12 weeks, whilst taking into account your personal level of fitness. There will also be a number of small-sided rugby game activities. You will also be encouraged to monitor your step count and follow a walking programme for the duration of the programme.

What are the benefits and risks of this study?

Possible benefits

The National Institute for Health Innovation
School of Population Health
Tamaki Campus
The University of Auckland
Private Bag 92019, Auckland, New Zealand
Telephone: 09 923 4730

Your participation will help researchers to see whether the RuFIT programme is effective as a healthy lifestyle programme. It is hoped that you will find participation in this study to be an enjoyable and rewarding experience.

Possible risks
There are no anticipated risks from taking part in this programme over and above the risks associated with taking part in a routine healthy lifestyle, which may include engaging in everyday physical activity (non-contact), modifying diet, and stopping smoking.

Will the information about me be kept confidential?

While it is unlikely that you may be identified, total confidentiality cannot be guaranteed. Further, as there will be other men taking part in the study, you will not be an anonymous participant.

The study files and all personal information that you provide will be strictly confidential. No material that could identify you will be used in any reports on this study. The National Institute for Health Innovation stores and backs up data either on The University of Auckland owned servers or on cloud services operated by a vendor with whom The University of Auckland have a contractual relationship. All computer records are password protected and paper records stored in a secure storage area. All future use of the information collected will be controlled in accordance with the Privacy Act, 1993. Any hard copy data will be kept in a locked cabinet in the Principal Investigator's office and kept separately from any other data.

During the study only the RUFIT-NZ researchers and study monitors will have direct access to your information. This access will only be to check the accuracy of the information collected for the study and the information will remain confidential.

What are my rights?

Your participation is entirely voluntary (your choice). You may withdraw from the study at any time up to the point of the 1-year data collection, without giving a reason. However, the information collected about you up to the point when you withdraw may continue to be used.

What if something goes wrong?

If you were injured in this study, which is unlikely, you would be eligible for compensation from ACC just as you would be if you were injured in an accident at work or at home. You will have to lodge a claim with ACC, which may take some time to assess. If your claim is accepted, you will receive funding to assist in your recovery.

What happens after the study?

When all participants have completed the study, the data will be analysed and published. At the earliest, this will be mid-2020. We will then notify you of the results by email. We may be asked to submit individual participant data to a clinical trials register in order to have the results published in a well-known journal. If we were required to submit data to such a register, you would not be able to be identified.

The National Institute for Health Innovation
School of Population Health
Tamaki Campus
The University of Auckland
Private Bag 92019, Auckland, New Zealand
Telephone: 09 923 4730

We will keep your information for 6 years after the study is completed.

Your data may be used in a study called a meta-analysis in the future. This type of study collects individual participant information from other parenting intervention trials. If we do share your data for such a study, you would not be able to be identified.

Who do I contact for more information or if I have concerns?

If you have any questions, concerns or complaints about the RUFIT-NZ study at any stage, you can contact:

Professor Ralph Maddison
Research Fellow, National Institute for Health Innovation, The University of Auckland, Private Bag 92019, Auckland 1142.
Phone: 08003676444
Email: r.maddison@auckland.ac.nz

Or you can contact the Head of Department:
Associate Professor Chris Bullen
Telephone: (09) 373-7999 x 84730.
Email: c.bullen@auckland.ac.nz

For any concerns regarding ethical issues, you may contact:

The Chair, The University of Auckland Human Participants Ethics Committee
The University of Auckland, Research Office, Private Bag 92019, Auckland 1142
Telephone 09 373-7599 ext. 83711. Email: ro-ethics@auckland.ac.nz

If you want to talk to someone who is not working on the study, you can contact a health and disability advocate on:

Phone: 0800 555 050
Fax: 0800 2 SUPPORT (0800 2787 7678)
Email: advocacy@hdc.org.nz

APPROVED BY THE UNIVERSITY OF AUCKLAND HUMAN PARTICIPANTS ETHICS COMMITTEE ON 20th September 2018 for 3 years, Reference Number 021888

Please keep this sheet for your information.
Thank you for taking the time to read about this study.

The National Institute for Health Innovation
School of Population Health
Tamaki Campus
The University of Auckland
Private Bag 92019, Auckland, New Zealand
Telephone: 09 923 4730

Consent Form

Rugby Fans In Training New Zealand (RuFIT-NZ)

This form will be held for a period of 6 years

Name of researchers: Professor Ralph Maddison, Dr Elaine Hargreaves, Dr Samantha Marsh, Dr Ihirangi Heke, Dr Stephen Kara, Dr Gerhard Sundborn, Dr Helen Elyes, Professor Kate Hunt, Professor Sally Wyke, Dr Cindy Gray, Professor Dave Lubans, Dr Yannan Jiang, Professor Nick Draper

I have read the Participant Information Sheet, and I have understood the nature of the research and why I have been selected. I have had the opportunity to use whanau/ family support or a friend to help me ask questions and understand the study and the questions have been answered to my satisfaction.

- I agree to take part in this study.
- I understand that taking part in this study is voluntary (my choice) and that I may withdraw from the study at any time.
- I agree that the information collected about me up to the point when I withdraw may continue to be used.
- I understand that my participation in this study is confidential and that no material, which could identify me personally, will be used in any reports on this study.
- I understand that information may be shared with other studies or registers but that no information that identifies me personally will be used.
- I understand that any data collected as part of this study will be stored securely at The University of Auckland, in accordance with the Privacy Act, 1993, and that all electronic data will be stored on the University of Auckland servers or cloud services.
- I understand the possible benefits and risk of the study.
- I know whom to contact if I have any questions about the study in general.

Declaration by participant [to be provided online]
I consent to take part in this study Yes ☐ No ☐

APPROVED BY THE UNIVERSITY OF AUCKLAND HUMAN PARTICIPANTS ETHICS COMMITTEE ON 20th September 2018 for 3 years, Reference Number 021888
