# Supplementary material for: Rugby Fans in Training New Zealand (RUFIT-NZ): protocol for a randomized controlled trial to assess the effectiveness and cost-effectiveness of a healthy lifestyle program for overweight men delivered through professional rugby clubs in New Zealand
Source: Trials. 2020 Feb 4;21:139. doi: 10.1186/s13063-019-4038-4 (PMC7001306; doi:10.1186/s13063-019-4038-4)
Supplement: Supplementary file 5 — Additional file 5. Award letter for study grant from the Health Research Council. [file 13063_2019_4038_MOESM5_ESM.pdf]

1 June 2018

Health Research Council of New Zealand

Professor Ralph Maddison
National Institute for Health Innovation
School of Population Health
The University of Auckland
Private Bag 92019
Auckland 1142

Dear Ralph

Project Application for Health Research Council Funding
HRC Reference: 18/513
Rugby Fans in Training: A Randomised controlled trial

The Health Research Council of New Zealand (HRC) has completed the assessment of all Project proposals for the 2018 funding round. I am very pleased to advise that your proposal has been successful. The Council has offered funding to the level set out on the attached draft contract Third Schedule Summary.

Please note that budgetary changes to your original application may necessitate a change in your research objectives. These changes, or other enquiries relating to the administrative aspects of your funding, should be directed to your Research Office. Once all changes have been agreed, contract documents will be sent to your Research Office for signing. A contract will not be formed until the HRC receives a completed "Staff Declaration - HRC Contracts Form" (available from your Research Office), amended objectives and milestones and any special conditions or requirements set out in the draft Third Schedule have been met. The *HRC Rules*, which form part of the contract, is available on the HRC website.

Some key conditions of the contract include best efforts to complete the proposed research, fulfilment of reporting requirements noting problems or delays as soon as they occur, changes or significant absences of key staff and significant changes to research objectives/ milestones. In your acceptance of this offer please indicate for our records whether an ethical approval is required for the planned research. Regular reporting aims to identify any issues or concerns as well as highlight positive outcomes of the research. Please let us know directly of any newsworthy impacts of our funding. Contract variations, such as time extensions, must be submitted to the HRC by your Research Office. All research reports can be now submitted on the HRC Gateway.

Your Research Office has been requested to return the draft Third Schedule within two weeks and accept contract offers by 31 July 2018. Unless your Research Office has received written authority from the HRC, your contract must commence no later than 1 October 2018. The funding may be withdrawn and returned to the HRC funding pool if this condition is not met.

Please note that the HRC will be making a media announcement about the outcome of this round in mid-June 2018. Media activities initiated by your institution may follow the HRC's announcement but must not be before this announcement. This includes posting any result details on your websites. Please contact the HRC if you would like us to provide comment for your institution's media funding announcements as we would be happy to do so.

Some of the points raised during the assessment of your application are outlined in the enclosed Review Summary. If you wish to discuss the result of your application please address your enquiry, in the first instance, to your host institution and request that they write to Dr Deming Gong, Manager Research Investment -Contracts, at the HRC.

Note that all investigators receiving contract funding from the HRC must make themselves available, as reviewers or assessing committee members whenever possible. Please update your HRC Gateway profile to nominate yourself for HRC assessing committee membership.

I would like to add a personal note of congratulations on your success and I look forward to hearing of the progress and outcomes of your research.

Yours sincerely

Professor Kathryn McPherson
Chief Executive

enc Review Summary
Draft Third Schedule
Draft Fourth Schedule

c.c. The University of Auckland Research Office

SAC Review Summary: Projects

**Funding Round**	AFR	**Application Type**	Project
**Reference**	18/513	**Applicant**	Maddison
**Title of Research**	Rugby Fans in Training: A Randomised controlled trial		

With regard to the criteria for assessing and scoring research proposals:

1. **What issues were considered by the Science Assessing Committee as important enough to influence the scoring of this proposal? (200-300 words)**

- The grant was supported by a previous feasibility study. However, it was not clear whether the intervention was still ongoing or not.
- The community focus of the grant was a strength of the application, including the ability to engage Māori and Pacific participants. The novelty of rugby club involvement was noted.
- The committee had concerns about the possible contamination from controls becoming active; how this would be handled was not fully described in the application.
- The committee was not convinced of the rationale for only recruiting men and suggested consideration be given to recruitment of women.

2. **Other Comments (please also include specific biostatistical feedback if not captured above)**

- Inclusion of discussion about the green script would have been helpful.

THIRD SCHEDULE SUMMARY - RESEARCH ACTIVITY DETAILS AND FUNDING

Host: The University of Auckland

Contract Type: .Project

Contract Number: 18/513

First Named Investigator Professor Ralph Maddison

Named Investigators: Dr Elaine Hargreaves, Dr Samantha Marsh, Dr Justin Ihirangi Heke, Dr Stephen Kara, Dr Gerhard Sundborn, Dr Yannan Jiang, Dr Helen Eyles, Professor Sally Wyke, Professor Kate Hunt, Dr Cindy M Gray, Professor David Lubans

DRAFT

Title: Rugby Fans in Training: A Randomised controlled trial

Proposed Start Date: 1/08/2018 **Completion Date:** 31/07/2021 **Term:** 36

Actual Start Date: ....

Organisations Sharing in Funding:

Reporting Dates: Annually on the anniversary of the grant plus 1 month

Budget Note: Incl. $154,585 funds for subcontract with University of Otago, to be released at contract commencement. Three months payment retained subject to satisfactory end of contract report

Budget Outline (GST Exclusive)

	$
**Total Budget:**	1,199,266.00
**Administered by HRC:**	0.00
**Host Budget:**	1,199,266.00

Monthly Payment:

33,312.95

Payment Process:

monthly on the 20th day of the month

Key Personnel

Professor Ralph Maddison	0.10
Dr Yannan Jiang	0.10
Dr Helen Eyles	0.03
Dr Elaine Hargreaves	0.08
Professor David Lubans	0.03
Dr Cindy M Gray	0.03
Professor Kate Hunt	0.03
Dr Stephen Kara	0.03
Professor Sally Wyke	0.03
Dr Samantha Marsh	0.20
Dr Justin Ihirangi Heke	0.03
Dr Gerhard Sundborn	0.03
Data Manager	0.15
Research Assistant	1.00
Project Manager	0.40
**Total FTE:**	**2.27**

Personnel marked * have a time commitment only

DRAFT

FOURTH SCHEDULE - RESEARCH OBJECTIVES AND MILESTONES

Note that this page will form the basis of the contract for progress reports

#	Objectives
1	Study set up
2	Baseline measurements commenced
3	Participants recruited
4	Follow-up measures completed
5	Data cleaning and data lock
6	Statistical analysis completed
7	Economic analysis completed
8	Re-AIM analysis completed
9	Final report completed

Year # for Completion of Milestones for each Objective		
Year #	Milestones	Objective(s)
1	Study set completed	1,2
1	Recruit study staff for RCT	1,1
1	Train RUFIT-NZ Coaches	1
1	Submit protocol manuscript to Trials	1,2
2	Recruit 360 participants for RCT	3
3	Study data collected	4,5
3	Trial completed	6,7,8,9
3	Submission of results paper to Journal	1,2,3,4,5,6,7,9
3	Submission of mansucript on implementation to IJBNPA	8

Contract Number:18/513 Date Printed: 4-May-2018

DRAFT
